# Dr. Genese's Method of Treating Fractured Jaw

**Published:** 1883-05

**Authors:** D. Genese


### TREATMENT OF FRACTURED JAW.

TO THE EDITOR AMERICAN JOURNAL OF DENTAL SCIENCE.

*Dear Sir*:—Having been greatly troubled in properly articulating a compound fracture of the inferior maxilla, I devised the following plan, which resulted in a success. I therefore send you my *modus operandi*, hoping it may be of benefit to the members of the profession who meet with the same difficulty.

The most important consideration is a perfect model of the fracture, this is obtained by the following process.

Place the parts together and secure the joints of the fracture by ligatures if possible, then firmly close the jaws and secure in place by a broad bandage, covering the mental process to the angle of the jaw, fastening it firmly to the top of the head, make a bag of coarse linen three inches wide, soap the wrong side and fasten one end, fill two-thirds with plaster and take the impression of the entire lower maxilla ; when this is dry and hard the bandage may be removed, the plaster impression held under the jaw firmly, and a perfect impression of the lower teeth and alveolar border taken at once, and in its natural position. I then dry my model, and take a die and counter in metal, making a tin cap the desired thickness and strength, forming a half round cushion or edge of wax on the tin plate that will be occupied on the finished plate by a rim of soft rubber, preventing abrasion of the tissues. This splint has the advantage of holding the parts firmly and accurately in their natural position retaining the articulaticn. Also giving the patient a moderately free use of his upper teeth, the external plaster splint keeping the fractured parts firmly fixed in position with the internal one so that no motion can occur, even if the bandages are removed. To secure them the usual skull cap with four buckles and straps is used. In forming the rubber splint any space necessary may be left for the escape of the discharge by building the model up with plaster. The whole should be vulcanized at a low temperature for three to four hours to insure perfect vulcanization ; and avoiding absorption of the secretions while the soft rim must be formed in one piece to prevent its being pressed into the hard rubber part and weakening the plates.

By this means very little disfigurement takes place around the fracture as the contour is firmly maintained until complete union takes place. The patient after a while is able to adjust it without danger of a displacement, and less time is occupied in effecting a cure.

I am, Yours Truly,

D. GENESE, D. D. S.

